# Consequences of Data Loss on Clinical Decision-Making in Continuous Glucose Monitoring: Retrospective Cohort Study

**DOI:** 10.2196/50849

**Published:** 2024-07-31

**Authors:** Niala den Braber, Carlijn I R Braem, Miriam M R Vollenbroek-Hutten, Hermie J Hermens, Thomas Urgert, Utku S Yavuz, Peter H Veltink, Gozewijn D Laverman

**Affiliations:** 1 Biomedical Signal and Systems Faculty of Electrical Engineering, Mathematics And Computer Science University of Twente Enschede Netherlands; 2 Internal Medicine Ziekenhuisgroep Twente Almelo Netherlands

**Keywords:** continuous glucose monitoring, missing data, clinical decision-making, clinical targets, time below range, TBR, diabetes mellitus, data interpretation, clinical practice, data analysis, continuous glucose monitoring metrics, glucose, diabetes, diabetic, metrics, data loss, decision-making, decision support, missing values, data science

## Abstract

**Background:**

The impact of missing data on individual continuous glucose monitoring (CGM) data is unknown but can influence clinical decision-making for patients.

**Objective:**

We aimed to investigate the consequences of data loss on glucose metrics in individual patient recordings from continuous glucose monitors and assess its implications on clinical decision-making.

**Methods:**

The CGM data were collected from patients with type 1 and 2 diabetes using the FreeStyle Libre sensor (Abbott Diabetes Care). We selected 7-28 days of 24 hours of continuous data without any missing values from each individual patient. To mimic real-world data loss, missing data ranging from 5% to 50% were introduced into the data set. From this modified data set, clinical metrics including time below range (TBR), TBR level 2 (TBR2), and other common glucose metrics were calculated in the data sets with and that without data loss. Recordings in which glucose metrics deviated relevantly due to data loss, as determined by clinical experts, were defined as expert panel boundary error (ε_EPB_). These errors were expressed as a percentage of the total number of recordings. The errors for the recordings with glucose management indicator <53 mmol/mol were investigated.

**Results:**

A total of 84 patients contributed to 798 recordings over 28 days. With 5%-50% data loss for 7-28 days recordings, the ε_EPB_ varied from 0 out of 798 (0.0%) to 147 out of 736 (20.0%) for TBR and 0 out of 612 (0.0%) to 22 out of 408 (5.4%) recordings for TBR2. In the case of 14-day recordings, TBR and TBR2 episodes completely disappeared due to 30% data loss in 2 out of 786 (0.3%) and 32 out of 522 (6.1%) of the cases, respectively. However, the initial values of the disappeared TBR and TBR2 were relatively small (<0.1%). In the recordings with glucose management indicator <53 mmol/mol the ε_EPB_ was 9.6% for 14 days with 30% data loss.

**Conclusions:**

With a maximum of 30% data loss in 14-day CGM recordings, there is minimal impact of missing data on the clinical interpretation of various glucose metrics.

**Trial Registration:**

ClinicalTrials.gov NCT05584293; https://clinicaltrials.gov/study/NCT05584293

## Introduction

Improved glucose sensing techniques have led to the increased availability of continuous glucose monitoring (CGM) technology for patients with diabetes. These minimally invasive sensors measure the glucose concentration of the interstitial fluid every 1 or 5 minutes, representing the blood glucose concentration with an average delay of 10-12 minutes [1,2]. CGM provides insights into glucose concentration fluctuations throughout the day and enables comparisons over time using predefined glucose metrics. This offers better prevention of out-of-range values compared to glycated hemoglobin (HbA_1c_) measurements or traditional finger prick methods. Consequently, CGM has the potential to improve glycemic control and adherence to lifestyle and drug regimens [3-7]. Therefore, CGM devices have been included in clinical guidelines and standards of care for patients with type 1 and 2 diabetes [8].

Commonly used glucose metrics to assess glycemic control in clinical practice from CGM devices include time in range (TIR), representing the percentage of time spent within the glucose range of 70-180 mg/dL (3.9-10.0 mmol/L), time below range (TBR) in 2 levels, time above range (TAR) in 2 levels, glucose management indicator (GMI), and the coefficient of variation (CV). Although treatment targets are individualized, the general aim is to achieve a TIR of over 70% while minimizing TBR below 4% [9,10].

Incomplete data collection during CGM monitoring can affect the clinical interpretation of glucose metrics. Several factors such as loss of connectivity, sensor or reader malfunction, depleted battery, or delayed interaction with the device when data are only temporarily stored on the sensor, could lead to data loss. This data loss can introduce bias in the estimation of glucose metrics [11]. Evaluating the potential impact of data loss on clinical interpretations and determining acceptable levels of error is crucial for clinical decision-making [12].

The type and distribution characteristics of missing data are important when evaluating data loss. There are 3 types of missing data, they are, missing completely at random (MCAR), missing at random (MAR), and missing not at random (MNAR) [13-15]. MCAR implies no systematic differences between participants with complete and incomplete data. MAR occurs when missing values are independent of the missing variables, but the pattern of missing data is dependent on time. In the case of MNAR, valuable information is lost from the data and there is no general method to manage the missing data properly [16,17]. Consecutive missing data, or gaps, have a certain gap size and incidence in the data, which can be represented by a gap probability distribution. Missing data are characterized as MCAR when the gap probability follows an exponential decline, with minimal affecting analysis and outcomes. However, MAR patterns skew the research outcomes, as missing data are more prevalent at certain times than others. Therefore, insight into the gap probability distribution is essential for understanding the influence of missing data on desired outcomes [15,18-20].

Currently, the recommendation for reliable interpretation of CGM data is to evaluate either 10 consecutive days without data loss or 14 consecutive days with a maximum data loss of 30% [8,10,21]. However, these studies have not investigated the impact of deviations from missing data in common CGM metrics on clinical decisions for individual patients [12,22,23]. Furthermore, previous research has determined that at least 14-15 days of CGM data provide a good estimation of CGM metrics compared to monitoring every 3 months or HbA_1c_ [21,24]. These studies primarily focused on the correlation between CGM data and HbA_1c_, which does not give insight into the impact of missing data on the clinical interpretation of different CGM metrics. Additionally, it only reflects long-term glycemic control, overlooking the potential for assessing short-term variations afforded by CGM data. Therefore, this study aims to investigate the effects of data loss on glucose metrics in individual patient recordings and its influence on clinical decision-making.

## Methods

### Patient Inclusion and Data Collection

This study was performed in the Diabase cohort, which is a registry of adult patients with type 1 diabetes and those with type 2 diabetes who use CGM technology as part of their care. They are treated in Ziekenhuisgroep Twente (ZGT), a local hospital in the Netherlands (NCT05584293). The exclusion criteria were dependency on hemodialysis or inability to provide informed consent.

All patients included in the Diabase cohort between September 2020 and March 2022 were reported upon, which contains retrospective data from June 2016.

### Ethical Considerations

The study was performed in accordance with the Declaration of Helsinki, the guidelines of good clinical practice. The Medical Research Ethics Committees United (MEC-U) in Nieuwegein, the Netherlands (registration AW23.009/W20.197), reviewed and approved the protocol. Prior to participation, patients provided informed consent to collect their (retrospective) glucose sensors and to retrieve relevant patient information from electronic patient files (age, gender, HbA_1c_, and BMI).

For this study, deidentified data were provided by the Diabase cohort, ensuring the confidentiality and privacy of participant information. Participants did not receive financial compensation for their participation in this study, as it solely involved the collection of data readily available from standard practice.

### CGM Derived Clinical Metrics

We collected CGM data using FreeStyle Libre sensors, and the data were stored in LibreView (Abbott Diabetes Care). The sensors have a storage capacity of up to 8 hours of data and measure for 2 weeks.

Derived from the CGM data were the mean glucose, TIR (glucose between 70 and 180 mg/dL or 3.9 and 10.0 mmol/L; %), TBR (glucose <70 mg/dL or 3.9 mmol/L; %), TBR level 2 (TBR2; glucose <54 mg/dL or 3.0 mmol/L; %), TAR (glucose >180 mg/dL or 10.0 mmol/L; %), TAR level 2 (TAR2; glucose >250.2 mg/dL or 13.9 mmol/L; %), SD glucose (mmol/L), CV (%), GMI (mmol/mol), low blood glucose index (LBGI), high blood glucose index (HBGI), and the risk index (RI; Multimedia Appendix 1) [8,10,25-27].

### Data Processing and Analysis

**Figure 1:**
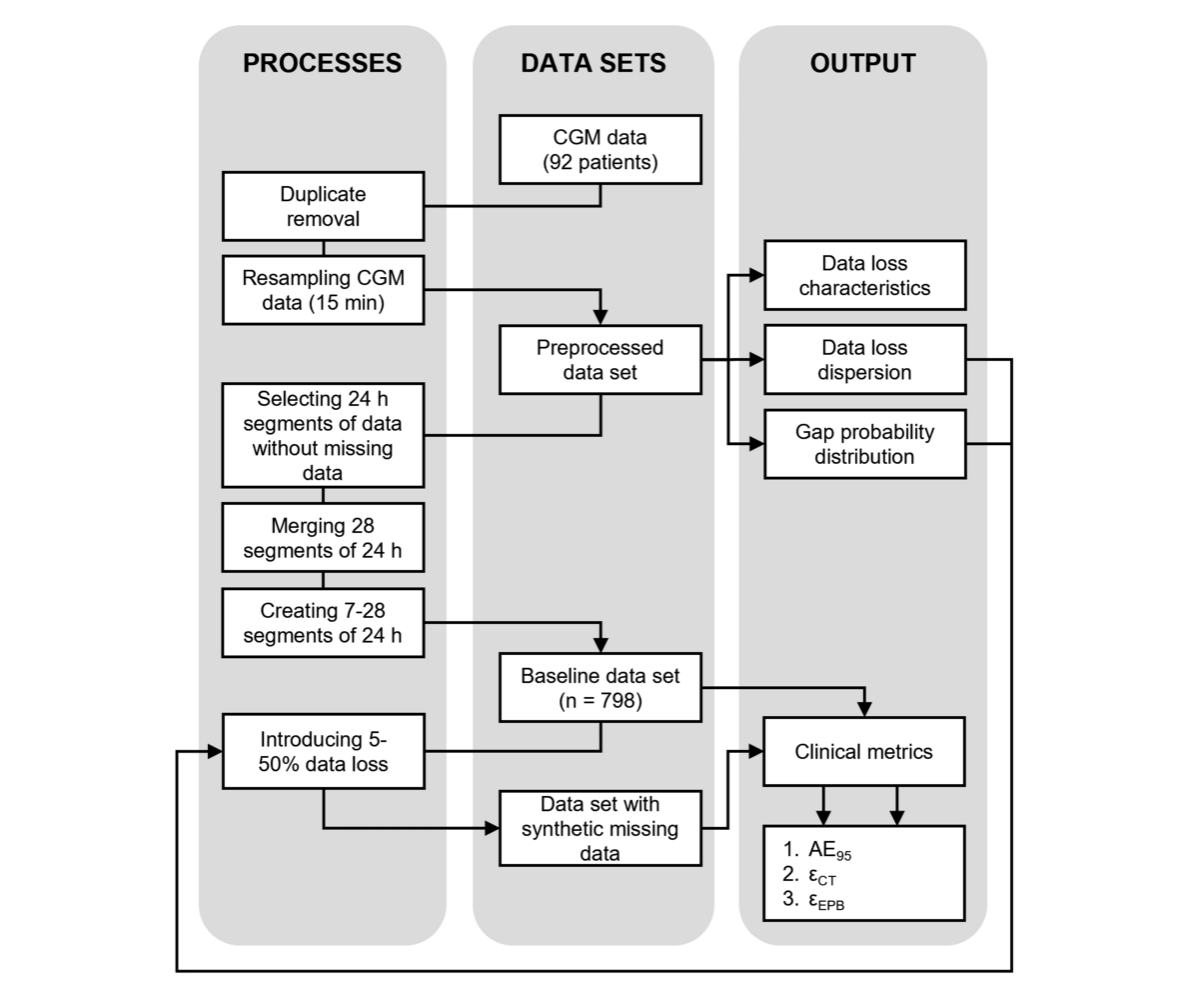
Data processing pipeline showing the data processing steps, the different data sets and the outputs. AE_95_: 95th percentile of absolute error; CGM: continuous glucose monitoring; ε_CT_: error clinical target; ε_EPB_: error expert panel boundaries.

Data from LibreView were extracted as a CSV file and processed in Python 3.9 (extension Spyder 5.3.1; Python Software Foundation). Figure 1 illustrates the data processing steps from the original CGM data into the various outputs used in this study. Data from the FreeStyle Libre sensor were recorded at intervals of 13-19 minutes (with an average every 15 minutes). First, duplicate data and data from multiple sources within 14 minutes were removed from the data set. Subsequently, data were resampled to 1 sample per 15 minutes using linear interpolation, starting at midnight. Interpolated data points were marked as missing when the time difference between the 2 nearest original data points exceeded 19 minutes. These steps resulted in the preprocessed data set.

From the preprocessed data set, the data loss characteristics were determined (illustrated in Figure 1). The percentage of missing data was calculated, and we determined the percentage of time that patients adhered to the data loss guidelines outlined in prior studies. These studies indicated that maintaining >70% of CGM data over the last 14 days or 10 days out of the 14 days strongly correlates with mean glucose, estimated HbA_1c_, TIR, and hyperglycemic metrics over a 3-month period. Consequently, our study adopts a 14-day measurement window with <30% data loss or a 10-day window with no data loss [8,21,24,28] The gap length and gap probability distribution were computed (Figure 1). To assess whether the missing data were MAR, we tested the fit of an exponential probability function to the gap probability distribution for gaps smaller than 96 samples [13]. The data loss dispersion over time was researched by comparing the average data loss per patient of the preprocessed CGM data set between various time periods—hours within the day, days in the week, business days (Monday to Friday) versus weekends, months, seasons, and days of the year.

It is important to consider the circadian rhythm of glucose metabolism during data analysis. Numerous studies have shown diurnal variations in glucose tolerance, insulin secretion, and peripheral insulin sensitivity, with poorer glycemic control observed in the evening and at night in healthy individuals [29,30]. To account for circadian rhythm, we constructed a baseline data set for each patient by combining 28 segments of 24-hour data without missing data (Figure 1). In cases that were where available, we merged multiple periods of 28 days per patient for further analysis, excluding patients with less than 28 segments of data. From the 28-day recordings, we created shorter recordings ranging from 7 up to 28 days.

Hereafter, data loss ranging from 5% to 50% was randomly introduced into the baseline data set in accordance with the gap distribution and data loss dispersion determined from the preprocessed data set. This means the gap distribution of the synthetic missing data is as close as possible to the gap distribution found over the whole population in the preprocessed data set. Also, synthetic data have higher chance of missing following the found data loss dispersion over time. Therefore, the synthetic missing data mimics the missing data as seen in day-to-day patient care.

### Absolute Errors

The CGM metrics were calculated for each recording in the baseline and synthetic missing data set, and compared for every recording. The absolute error (AE), the median AE (MedAE), and the 95th percentile of the AE (AE_95_) as a result of missing data were calculated for all CGM metrics (Figure 1).

### Clinical Target Errors (ε_CT_)

The clinical targets follow clinical guidelines—<4% TBR, <1% TBR2, >70% TIR, <25% TAR, <5% TAR2, <36% CV, and <53 mmol/mol GMI [9,10]. The percentage of recordings that surpassed the clinical target cut-off because of missing data was determined as the clinical target error (ε_CT_). This step is illustrated in Figure 1.

### Expert Panel Boundary Error (ε_EPB_)

A panel of experts consisting of a diabetes specialist nurse, a diabetes nurse, and a technical physician (a medical specialist in diabetes-related technology in health care) was interviewed. Each expert was interviewed individually to discuss clinically relevant changes in CGM metrics. During the interviews, CGM metrics TIR, TAR, TAR2, TBR, TBR2, GMI, and CV were discussed separately. The experts were instructed to consider a generic patient with diabetes and to evaluate each CGM metric separately. They were asked to identify when a change in the CGM metric would likely result in therapy alteration, indicating clinical relevance. A clinically relevant change can be dependent on the initial value of the CGM metric. For example, a change in TBR from 2% to 4% can have more impact than a change from 8% to 10%, even though the absolute change is the same. Therefore, the experts determined clinically relevant changes for various initial values of all CGM metrics (Multimedia Appendix 2). From these discussions, the strictest relevant change per CGM metric was selected as the expert panel boundary (Multimedia Appendix 2). The percentage of recordings that exceed the defined expert panel boundary due to data loss is defined as the expert panel boundary error (ε_EPB_), this step is depicted in Figure 1. When data loss resulted in an ε_CT_ or ε_EPB_ of more than 5%, we assumed that data loss had considerable influence on the CGM metric and should be interpreted with caution, consistent with the clinically significant criterion for a 5% increase in TIR [8].

### GMI Subgroup Analysis

To see whether missing data had a different influence on TBR, the recordings were divided into 3 GMI-based groups (<53, 53-64, and ≥64 mmol/mol), based on 3 commonly used HbA_1c_ categories as determined by the American Diabetes Association [31]. Hereafter, the MedAE, ε_CT_, and ε_EPB_ were calculated for TBR in the 3 GMI groups.

### Statistical Analysis

The median and IQR were calculated for the clinical and sensor data characteristics, that is, the recording length, data loss, and overall CGM metrics.

The fit between the gap distribution and the exponential probability density function was tested with a 2-sided Kolmogorov-Smirnov test and the sum of the squared error. Differences in data loss dispersion between several time periods were evaluated using the Kruskal-Wallis test with post hoc Dunn test and Bonferroni correction. Differences between the glucose metrics between the preprocessed data set and the baseline data set with 28 days were evaluated using Kruskal-Wallis test with Bonferroni correction. For these statistical analyses, the packages SciPy and scikit-posthocs in Python were used [32,33]. All data visualizations were made with the Python package Matplotlib [34].

Scatterplots were generated for CGM metrics from 14-day recordings with complete data versus those with 30% synthetic missing data. Additionally, histograms of the AE were constructed from 14-day recordings with 30% data loss in TBR and TBR2. The histogram bin widths were determined with Freedman-Diaconis rule. A P value <.01 was considered significant.

## Results

### Data and Patient Characteristics

Between September 2020 and March 2022, 92 patients using a FreeStyle Libre CGM device were included in the Diabase study. Descriptive statistics of the population can be found in Table 1.

**Table 1:** Characteristics of the population and their data used in this research.

	Values
Type 1 diabetes, n (%)	79 (95.8)
Type 2 diabetes, n (%)	13 (14.2)
Men, n (%)	47 (51)
Age (years), median (IQR)	52 (37.3-60.5)
BMI (kg/m^2^), median (IQR)	26.2 (23.1-29.4)
HbA_1c_^a^ (%), median (IQR)	7.6 (7.0-8.3)
HbA_1c_ (mmol/mol), median (IQR)	60 (53.3-67.0)
Recording length (days), median (IQR)	655 (499-925)
Data loss (%), median (IQR)	13.5 (6.3-35.6)
Time recordings had 0% data loss for 10 days (%), median (IQR)	0.0 (0.0-0.3)
Time recordings had <30% data loss for 14 days (%), median (IQR)	88.9 (63.0-98.7)

^a^HbA_1c_: glycated hemoglobin.

### Data Loss Statistics

The gap probability distribution of the preprocessed data set differs significantly from a fitted exponential probability density function (P=.002). This means the data loss is not completely random (Multimedia Appendix 3).

During the hours of 10-11 PM, 11 PM to midnight, and midnight to 1 AM, a higher data loss of 35.8%, 43.3%, and 35.3%, respectively, was observed compared to other hours of the day with an average data loss of 10.4% (IQR 2.7%-30.5%; P<.001). Therefore, to mimic the unequal dispersion we created synthetic missing data following the probability of missing data during the day. No significant differences in data loss were found between other time periods.

### Baseline Data Set

Among the 92 patients, 84 (91%) patients had sufficient data to construct a data set of 28 segments with complete 24-hour segments of data. These 84 patients contributed to a total of 798 recordings over 28 days, forming the baseline data set. Each patient provided median of 7.0 (IQR 3.8-14.0) in 28-day recordings. The median (IQR) for TIR, TBR, TBR2, TAR, and TAR2 of all 28-day recordings were 60.3% (53.2%-67.8%), 3.3% (1.4%-5.8%), 0.3% (0.0%-1.0%), 35.4% (27.4%-43.3%), and 9.4% (5.5%-13.9%), respectively. No significant differences were found between all the clinical metrics of the preprocessed data and the 28-day baseline data set, indicating that the selected baseline data set reflects the original data.

### Expert Panel

The experts unanimously agreed that the most important metrics were TBR and TBR2, resulting in strict expert panel boundaries. In the TBR range of 0%-4% and the TBR2 range of 0%-5%, a difference of 1% was deemed clinically relevant (Multimedia Appendix 2). However, as the TBR increased, the experts allowed for higher changes, accepting a maximum of 5% for TBR and 10% for TBR2 when the initial value was 100%. Expert panel boundaries for the remaining glucose metrics can be found in Multimedia Appendix 2.

### CGM Metrics Without and Those With Synthetic Data Loss

In the baseline data set, 734 (92.0%), 408 (51.1%), 796 (99.8%), and 767 (96.1%) of the total (N=798) 7-day recordings contained TBR, TBR2, TAR, and TAR2, respectively. For 28-day recordings, this is 798 (100%), 611 (76.6%), 798 (100%), and 790 (99.0%) for TBR, TBR2, TAR, and TAR2, respectively.

**Figure 2:**
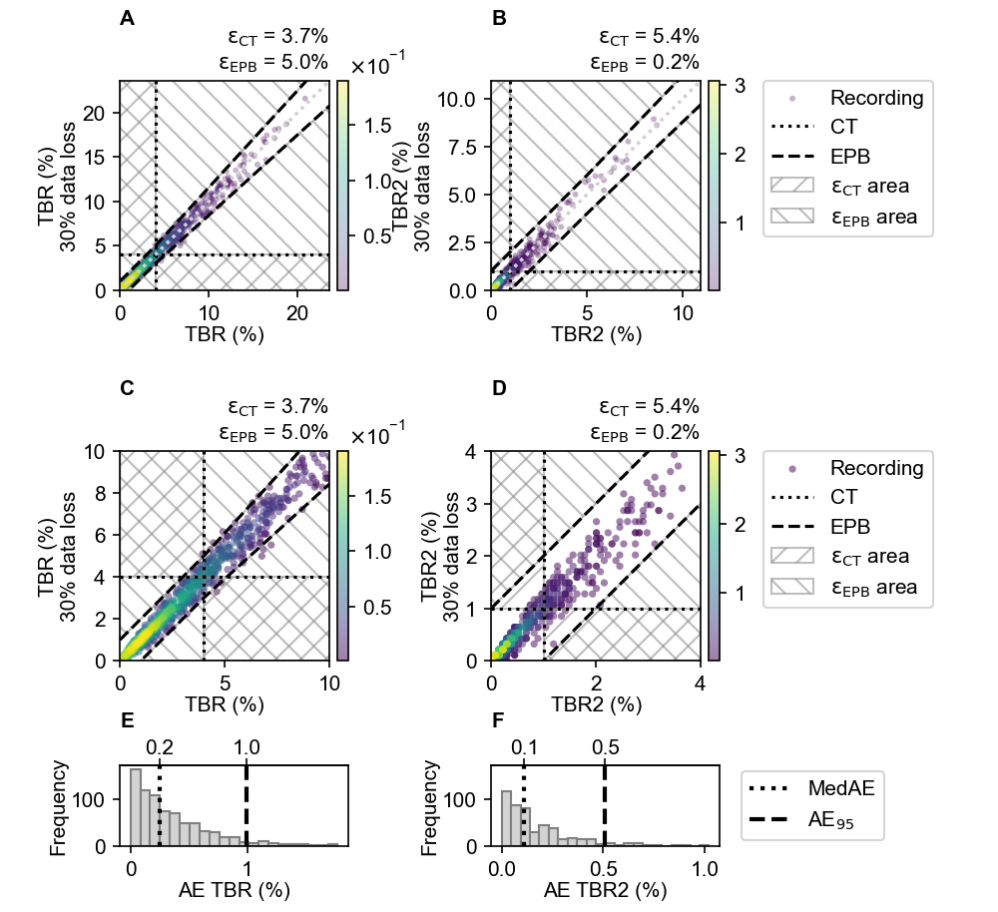
(A) The density scatterplots of the TBR and (B) TBR2 indicates the relation between the 14-day-long original data without missing data (horizontal axis) and the data with 30% data loss (vertical axis). More detail of the critical areas of TBR and TBR2 are illustrated in (C) and (D). The color bar indicates the Gaussian kernel-density estimate of the recordings. The black dashed lines represent the expert panel boundaries, and the horizontal and vertical black dotted lines represent the clinical targets. Values falling outside the clinical target and expert panel boundary, in the hatched areas, are labeled as errors (ε_CT_ and ε_EPB_). (E) The histograms show the AE (%) with the median AE (MedAE) and AE_95_ as a result of 30% data loss of the TBR and (F) TBR2. AE: absolute error; AE_95_: 95th percentile of AE; CT: clinical target; EPB: expert panel boundary; MedAE: median absolure error; TBR: time below range; TBR2: time below range level 2; ε_CT_: clinical target errors; ε_EPB_: expert panel boundary errors.

The scatterplots of Figure 2A represent the relation between the original 14-day data of TBR and TBR2, and with 30% data loss, per individual recording. These figures show that 30% data loss in these metrics results in small deviations from the true value with data loss, as all data points are close to the identity line. More recordings fell into the ε_EPB_ area for TBR, as the expert panel boundary was stricter compared to TBR2. The histograms of Figure 2B show the AE, MedAE, and AE_95_ for these CGM metrics as a result of 30% data loss in a 14-day recording. The MedAE in the histograms is small, indicating that the majority of errors are small. However, the histograms also display a long tail, indicating that there are instances where the AE is larger than the MedAE.

**Figure 3:**
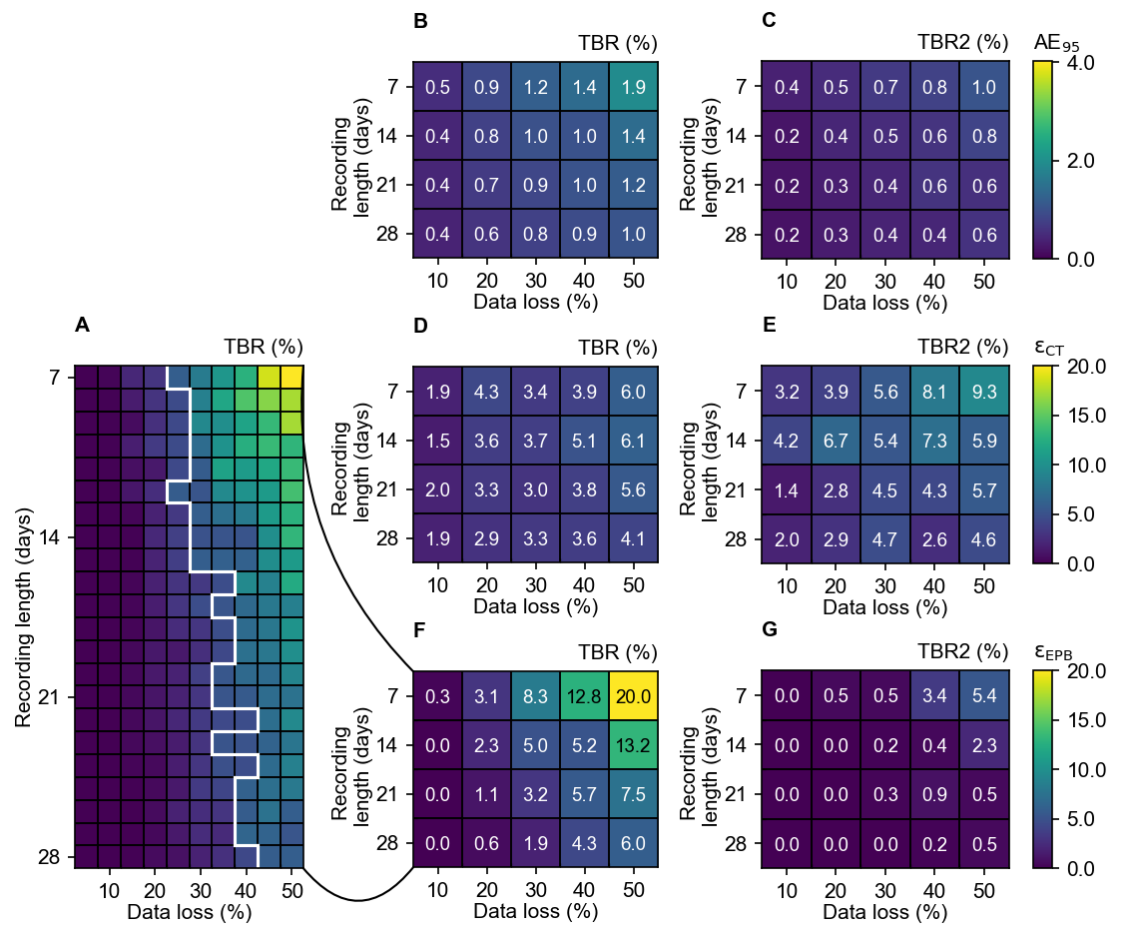
(A) The ε_EPB_ of 5%-50% data loss in recordings (horizontal axis) of 7-28 days (vertical axis) of TBR. The white line indicates the boundary where the ε_EPB_ exceeds 5%. (B,C) show the AE_95_, (D,E) the clinical target errors (ε_CT_), and (F,G) the ε_EPB_ of TBR and TBR2 respectively of 10%, 20%, 30%, 40%, and 50% data loss in recordings of 7, 14, 21, and 28 days. The color bar indicates the errors in percentages. AE_95_: 95th percentile of the absolute error; TBR: time below range; TBR2: time below range level 2; ε_EPB_: expert panel boundary errors; ε_CT_: clinical target errors.

Figure 3 shows the values of AE_95_, ε_CT_, and ε_EPB_ for the CGM metrics TBR and TBR2, with missing data increasing from 5% to 50% over a period of 7 to 28 days. This analysis includes only the recordings that had TBR episodes. As expected, the AE_95_, ε_CT_, and ε_EPB_ increase as the percentage of data loss increases. When more days are available, the influence of data loss is reduced (Figure 3A). Applying the current guidelines of 30% data loss for a 14-day recording period, we observed an AE_95_ of 1.0% for TBR and 0.5% for TBR2. The corresponding ε_CT_ values were 29 out of 786 (3.7%) recordings for TBR and 28 out of 522 (5.4%) recordings for TBR2 and the ε_EPB_ were 28 out of 786 recordings (5.0%) for TBR 1 out of 522 recordings and 0.2% for TBR2. These findings suggest that the impact of missing data on CGM metrics can vary significantly depending on the specific metric and recording period used.

For a 14-day measurement period with 30% data loss, the AE_95_ was 2.4%, 1.3%, and 1.0%, ε_CT_ was 17 (2.1%), 23 (2.9%), and 14 (1.8%) and ε_EPB_ was 0 (0.0%), 0 (0.0%), and 0 (0.0%) for TIR, CV, and GMI, respectively (Figures S1 and S2 in Multimedia Appendix 4). For TAR and TAR2 the ε_EPB_ was 0.0% for all cases (Figure S3 in Multimedia Appendix 4).

TBR and TBR2 have the highest errors, especially with greater data loss in shorter recordings, with a maximum ε_EPB_ of 147 out of 736 (20.0%) of the recordings for TBR and 22 out of 408 (5.4%) of the recordings for TBR2, compared to a maximum of 5 out of 798 (0.6%) for TIR, CV, and GMI. In 2 (0.3%) of the 14-day recordings, the TBR value went from 0.2% to 0.0%, resulting in its disappearance due to data loss. Similarly, the TBR2 metric disappeared in 32 (6.1%) recordings in the 14-day recordings with 30% data loss. The median TBR2 value of the original recordings was 0.07% (IQR 0.07%-0.15%). No other metrics disappeared due to data loss.

For the SD, LBGI, HBGI, and RI, only the AE_95_ was available. The overall AE_95_ was low, with 0.1 for SD, 0.2 for LBGI, and 0.6 for HBGI and RI. The highest AE_95_ of 0.8% was observed for the HBGI and RI metrics in a 7-day recording with 50% data loss (Figure S4 in Multimedia Appendix 4).

### GMI Subgroup Analysis

**Figure 4:**
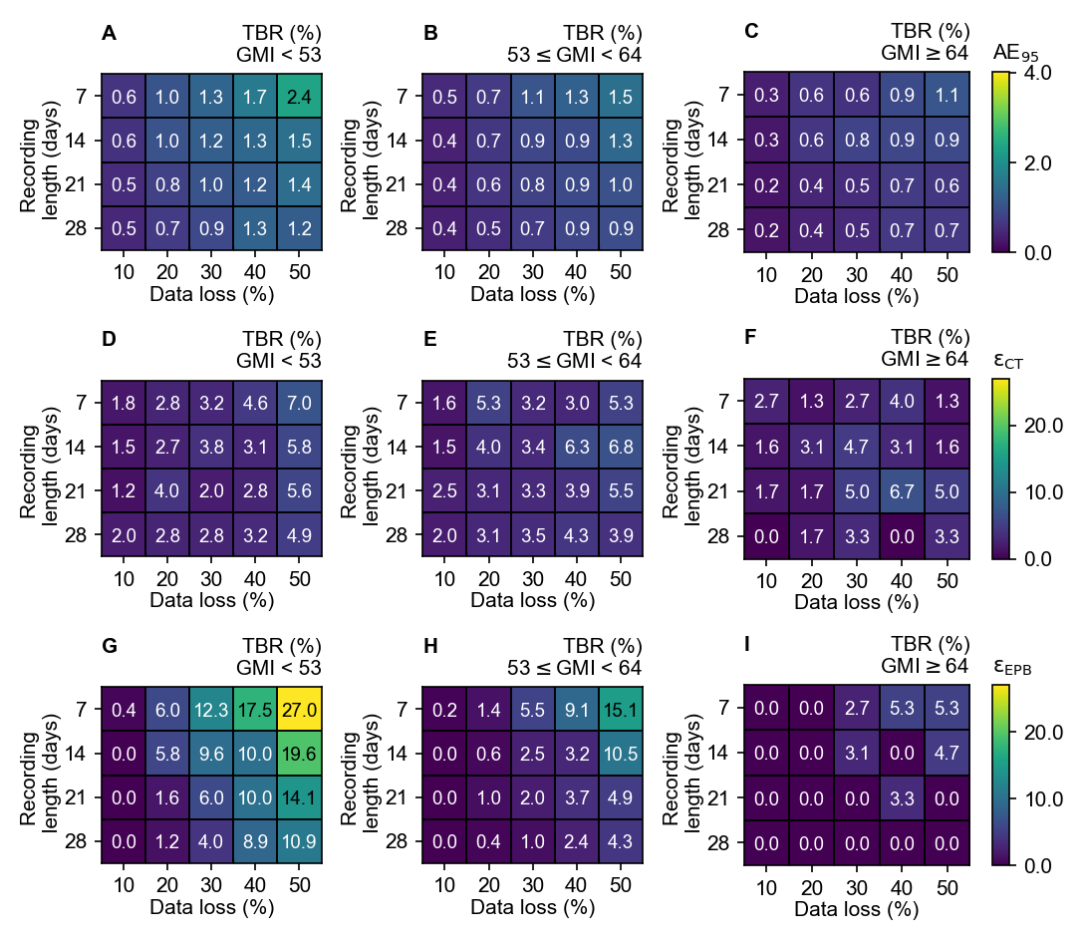
(A-C) The AE_95_ are shown, (D-F) the ε_CT_, and (G-I) the ε_EPB_ of TBR within 3 GMI groups of <53 mmol/mol, 53 mmol/mol≤ GMI <64 mmol/mol, and GMI ≥64 mmol/mol. These panels illustrate the impact of 10%, 20%, 30%, 40%, and 50% data loss in recordings of 7, 14, 21, and 28 days. The color bar indicates the errors in percentages. AE_95_: 95th percentile of the absolute error; GMI: glucose management indicator; TBR: time below range; ε_CT_: clinical target errors; ε_EPB_: expert panel boundary errors.

The recordings were divided into 260 (32.6%) low GMI (<54 mmol/mol), 445 (55.8%) moderately elevated (53-64 mmol/mol), and 93 (11.7%) elevated GMI recordings (≥64 mmol/mol). Figure 4 shows the values of AE_95_, ε_CT_, and ε_EPB_ for TBR, with missing data increasing from 5% to 50% over a period of 7 to 28 days for the GMI subgroups. The error caused by missing data is highest in the low GMI group (Figures 4A, 4D, and 4G). The AE_95_, ε_CT_, and ε_EPB_ are 1.2%, 10 (3.8%), and 25 out of recordings 260 (9.6%), respectively, for a recording of 14 days with 30% data loss. This means that for 9.6% of the recordings a clinical expert would see a relevant change.

## Discussion

### Principal Findings

This study provides a thorough analysis of missing data’s impact on real-world CGM recordings for patients with diabetes. By merging data analysis and expert evaluations, it assesses the clinical implications of missing data on CGM metrics, improving our understanding of its practical effects and challenges. Findings indicate that a minimum of 14 days of glucose data collection with no more than 30% missing data suffice for clinical decision-making, ensuring adequate patient care.

The key finding of this study is that the interpretation of TBR metrics is more vulnerable to greater clinical consequences due to missing data compared to other metrics. The results indicate that up to 30% data loss in a 14-day recording results in a misinterpretation of the glucose metric in 5.0% of the time. Therefore, it should be realized that 30% data loss over a 14-day measurement will occasionally lead to false clinical interpretations of TBR. Furthermore, when analyzing the data by GMI levels, it is notable that in 9.6% of recordings featuring low GMI levels, a clinical expert would observe a relevant change due to missing data on TBR. These findings underscore the substantial impact of missing data on TBR, emphasizing the importance of cautious interpretation in clinical practice.

Similarly, for TBR2, 14-day recordings with 30% data loss resulted in misinterpretation in 0.2% of cases, and the complete disappearance of TBR2 episodes occurred in 6.1% of the cases. One might, therefore, suggest that severe hypoglycemic episodes would be missed, which can have potentially serious clinical consequences as symptoms might not always be recognized when they occur during nighttime [35]. However, this complete loss of TBR2 episodes occurs only when the actual TBR2 value is below the clinical target of 1%, and thus, would not have required any action. In contrast, missing data had limited influence on the clinical interpretation of other glucose metrics such as TIR, CV, GMI, TAR and TAR2. This limited influence was partly caused by broader expert panel boundaries for these metrics compared to TBR and TBR2. The expert panel accepted errors of 1%-5% for TBR2 and 1%-10% for TBR, while the accepted errors for TIR, TAR, and TAR2 ranged between 5% and 10%. When TBR is not a primary concern, 14 days of CGM data can be interpreted with 50% missing data. Even measurements shorter than 14 days could be used, but the representativeness of the data for long-term glucose values will diminish, and the correlation of the metrics with HbA_1c_ may decrease [21].

Based on the findings of this study, we propose several recommendations. First, we support the current recommendation of having a minimum of 70% data available in a CGM recording of 14 days. However, health care practitioners should be aware that there is still a chance of misinterpreting TBR. The guideline of a 10-day CGM recording with no data loss might be impractical, as the real-life CGM data analyzed in this study rarely met this criterion [8]. Therefore, our second recommendation is to use a measurement period of at least 14 days, which aligns with the durability of the current sensors. However, this result should be validated by other studies with different glucose sensors.

In this study, the expert panel boundaries are an important feature that may contribute to current clinical guidelines as they give insight into when CGM metrics result in clinically relevant changes due to data loss. The current clinical targets for most CGM metrics are valuable in the clinical setting to serve as goals for optimal glucose management. However, the targets are a limited tool for determining when a patient has a clinically relevant improvement or deterioration of their glucose management. When assessing the clinical consequences, the clinical target may not always be useful because significant changes due to data loss may not result in a different categorization if the initial value is already far from the target. However, such changes may still be clinically relevant.

In the literature, the CGM metrics SD, LBGI, HBGI, and RI are defined [21]. However, there are currently no targets defined for these metrics. Consequently, the expert panel was unable to determine such a boundary for these metrics. Nevertheless, AE_95_ can provide insight into the error size, but not indicate the impact on clinical decision-making. Previous studies investigating the influence of missing data on glycemic variability metrics reported small MedAEs [12,22]. This corresponds to our findings of small MedAEs, suggesting that even with excessive data loss, the CGM metrics would not be altered significantly. However, the AE_95_ is in some cases relatively high, indicating that in 5% of the population data loss can have quite significant clinical consequences. Therefore, we reported on AE_95_ instead of MedAE in this study.

The data loss in the real-world CGM data was MNAR, as the gap probability distribution did not follow an exponential function. Next to that, the chance of missing data during the night (10 PM-1 AM) was higher. MNAR data loss has a profound impact on the effects of missing data and synthetic data loss cannot be applied arbitrarily. Therefore, in this research, we mimicked these characteristics of data loss to create a simulation that closely resembled the actual missing data experienced by patients using CGM devices in everyday life.

### Limitations

Several potential limitations of this study need to be addressed. In clinical practice, all CGM metrics are typically evaluated together rather than individually which was done to determine the clinical expert boundaries. Additionally, these metrics will be reviewed at every clinical visit, further reducing the actual risk that missing data will cause a change in treatment. Another potential limitation of our study is the small number of experts involved in defining the boundaries for CGM metrics. The subjectivity and potential biases inherent in expert input can pose challenges. Furthermore, the cut-off to accept an ε_EPB_ at 5% was chosen arbitrarily and choosing a different cut-off could influence the interpretation of the results. It is important to note that defining different boundaries than those presented in this study could influence the observed errors.

It should also be considered that this research has been performed on data measured only with the FreeStyle Libre 1 sensor, and some findings might differ for other devices. For example, the 8-hour data storage capacity may explain why more data are lost during the early night (10 PM-1 AM), which is a critical time for glucose measurements [36]. However, the presented research methodology can be easily adopted and applied to other CGM devices or populations. The results presented in our study were obtained from patients with diabetes, predominantly type 1 diabetes, who were treated in a hospital in the Netherlands. Differences in patient demographics, disease progression, and device accuracy could impact the applicability of findings across diverse populations. Therefore, caution should be exercised when extrapolating these results to other populations. However, the average HbA_1c_ value of 7.6% (IQR 7.0%-8.3%; 60.0 mmol/mol, IQR 53.3-67.0 mmol/mol) was comparable with a large cohort from Germany and Austria, with a mean HbA_1c_ of 7.8% (IQR 6.9%-8.9%; 62, IQR 52.0-74.0 mmol/mol), suggesting some level of generalizability [37]. Furthermore, the presented methodology can be applied to investigate the consequences of missing data in other diabetes populations using CGM.

### Methodological Decisions

Some noteworthy methodological decisions were made in this study. First, we decided to include all the available 24-hour windows, thus including multiple 28-day recordings per patient in the data set. With this approach, there were no significant differences in CGM metrics between the preprocessed data with the baseline data set, validating the inclusion of multiple recordings. Second, not all patients used their CGM device continuously. Patients may have periods of several months where they did not use a CGM device. We marked this as missing data, which may have led to a potential overestimation of the reported data loss, compared to day-to-day CGM use. Third, we decided to create recordings of 7 to 28 days to study a range of CGM measurement lengths. The studies of Riddlesworth et al [21] and Xing et al [24] suggest that at least 14-15 days of CGM data provide a good estimation of CGM metrics compared to monitoring every 3 months or HbA_1c_. Also, Akturk et al [23] state that while the optimal recording length depends on the size of the gaps, duration of 14 days generally proves to be adequate. Using measurements of 7-28 days, we can ensure that an adequate measurement duration was covered.

### Future Research

Future research should focus on including a heterogeneous population of patients with type 1, type 2, and other subtypes of diabetes. Next to that, more commonly used sensors should be added to the analysis to give a more generalizable result. Finally, the expert panel should be expanded and implemented similarly to the Parkes and Clarke error grids, involving a larger and more diverse panel of experts, to enhance the reliability and generalizability of the established boundaries [38,39].

### Conclusions

To conclude, our aim was to examine the impact of data loss on glucose metrics within individual patient recordings from continuous glucose monitors and its implications for clinical decision-making. Through integrated data analysis and expert evaluations, we underscore the importance of comprehending missing data’s clinical consequences and recommend a maximum of 30% missing data in 14-day CGM recordings to enhance accurate interpretation and glucose management, acknowledging the possibility of misinterpreting TBR even with this threshold. For reliable interpretation of TBR in recordings with a low GMI, data loss should be below10%. Further research is needed to explore the consequences of missing data in diverse populations using various CGM devices, emphasizing the importance of comprehensive data collection for optimal glucose management and clinical decision-making.

## Appendix

Multimedia Appendix 1 Formulas of the clinical continuous glucose monitoring (CGM) metrics.

Multimedia Appendix 2 Expert panel boundaries and the clinically relevant change, using the initial metric value as a baseline, for the seven most commonly used clinical continuous glucose monitoring (CGM) metrics, along with their respective clinical targets.

Multimedia Appendix 3 Description of the method and results of fitting the gap probability distribution of preprocessed continuous glucose monitoring (CGM) data with the exponential and other common probability mass functions.

Multimedia Appendix 4 Density scatterplots for time in range (TIR) and coefficient of variation (CV) and the error plots for glucose metrics TIR, CV, glucose management indicator (GMI), time above range (TAR), TAR level 2 (TAR2), SD, low blood glucose index (LBGI), high blood glucose index (HBGI), and risk index (RI).

## Abbreviations

ε_CT_: clinical target error
ε_EPB_: expert panel boundary error
AE: absolute error
AE_95_: 95th percentile of the absolute error
CGM: continuous glucose monitoring
CV: coefficient of variation
GMI: glucose management indicator
HbA_1c_: glycated hemoglobin
HBGI: high blood glucose index
LBGI: low blood glucose index
MAR: missing at random
MCAR: missing completely at random
MEC-U: Medical Research Ethics Committees United
MedAE: median absolute error
MNAR: missing not at random
RI: risk index
TAR: time above range
TAR2: time above range level 2
TBR: time below range
TBR2: time below range level 2
TIR: time in range
ZGT: Ziekenhuisgroep Twente

## Data Availability

The data sets generated and analyzed during this study are not publicly available due to the ongoing inclusion of subjects but are available from the principal investigator (TU) on reasonable request.
