# Supplementary material for: Consequences of Data Loss on Clinical Decision-Making in Continuous Glucose Monitoring: Retrospective Cohort Study
Source: Interact J Med Res. 2024 Jul 31;13:e50849. doi: 10.2196/50849 (PMC11325125; doi:10.2196/50849)
Supplement: Multimedia Appendix 1 [file ijmr_v13i1e50849_app1.docx]

| Clinical CGM Metrics | Formula |
| --- | --- |
| Mean glucose (mmol/L), $\bar{Gluc}$ | $\bar{Gluc}=\frac{1}{n}\sum_{i=1}^{n} Gluc(i)$ |
| Time in range (%), TIR | $Gluc(i)\left\{ \begin{aligned} 3.9\leq Gluc\left( i \right)\leq10.0;IR\left( i \right)=1 \\ otherwise;IR\left( i \right)=0 \end{aligned} \right.$ |
|  |  |
|  | $TIR=\frac{1}{n}\sum_{i=1}^{n} IR\left( i \right)*100\%$ |
|  |  |
| Time below range (%), TBR | $Gluc(i)\left\{ \begin{aligned} Gluc\left( i \right)<3.9;BR\left( i \right)=1 \\ otherwise;BR\left( i \right)=0 \end{aligned} \right.$ |
|  |  |
|  | $TBR=\frac{1}{n}\sum_{i=1}^{n} BR\left( i \right)*100\%$ |
|  |  |
| Time below range level 2 (%), TBR2 | $Gluc(i)\left\{ \begin{aligned} Gluc\left( i \right)<3.0;BR2\left( i \right)=1 \\ otherwise;BR2\left( i \right)=0 \end{aligned} \right.$ |
|  |  |
|  | $TBR2=\frac{1}{n}\sum_{i=1}^{n} BR2\left( i \right)*100\%$ |
|  |  |
| Time above range (%), TAR | $Gluc(i)\left\{ \begin{aligned} Gluc\left( i \right)>10.0;AR\left( i \right)=1 \\ otherwise;AR\left( i \right)=0 \end{aligned} \right.$ |
|  |  |
|  | $TAR=\frac{1}{n}\sum_{i=1}^{n} AR\left( i \right)*100\%$ |
|  |  |
| Time above range level 2 (%), TAR2 | $Gluc(i)\left\{ \begin{aligned} Gluc\left( i \right)>13.9;AR2\left( i \right)=1 \\ otherwise;AR2\left( i \right)=0 \end{aligned} \right.$ |
|  |  |
|  | $TAR2=\frac{1}{n}\sum_{i=1}^{n} AR2\left( i \right)*100\%$ |
|  |  |
| Standard deviation glucose (mmol/L), SD | $SD=\sqrt{\frac{\sum_{i=1}^{n} \left( Gluc(i)-\bar{Gluc} \right)}{n}}$ |
|  |  |
|  |  |
|  |  |
| Coefficient of variation (%), CV | $\frac{SD}{\bar{Gluc}}*100\%$ |
|  |  |
|  |  |
|  |  |
| Glucose management indicator (mmol/mol), GMI | $GMI=12.71+4.70587\cdot\bar{Gluc}$ |
|  |  |
|  |  |
|  |  |

| Clinical CGM Metrics | Formula |
| --- | --- |
| Low blood glucose index, LBGI | $f\left( Gluc\left( i \right) \right)=1.794*{\log\left( Gluc(i) \right)}^{1.026}-1.861$ |
|  | $rl\left( Gluc(i) \right)=\left\{ \begin{aligned} f\left( Gluc(i) \right)\geq0;0 \\ f\left( Gluc(i) \right)<0;10\cdot f\left( Gluc(i) \right)^{2} \end{aligned} \right.$ |
|  | $LBGI_{uncorrected}=\frac{1}{n}\sum_{i=1}^{n} rl\left( Gluc(i) \right)$ |
|  | $LBGI=1.0199*LBGI_{uncorrected}+0.6251$ |
| High blood glucose index, HBGI | $rh\left( Gluc(i) \right)=\left\{ \begin{aligned} f\left( Gluc(i) \right)>0;10\cdot f\left( Gluc(i) \right)^{2} \\ f\left( Gluc(i) \right)\leq0;0 \end{aligned} \right.$ |
|  | $HBGI=\frac{1}{n}\sum_{i=1}^{n} rh\left( Gluc(i) \right)$ |
| Risk index, RI | $RI=LBGI+HBGI$ |

Gluc: glucose measurements, expressed in mmol/L; n: total number of glucose samples; f: function; rl: risk function low; rh: risk function high
