# Supplementary material for: Consequences of Data Loss on Clinical Decision-Making in Continuous Glucose Monitoring: Retrospective Cohort Study
Source: Interact J Med Res. 2024 Jul 31;13:e50849. doi: 10.2196/50849 (PMC11325125; doi:10.2196/50849)
Supplement: Multimedia Appendix 2 [file ijmr_v13i1e50849_app2.docx]

Expert panel boundaries and the clinically relevant change, using the initial metric value as a baseline, for the seven most commonly used clinical CGM^a^ metrics^b^, along with their respective clinical targets.

| Clinical CGM metrics, clinical targets (unit) | Expert panel boundaries | |
| --- | --- | --- |
|  | Initial metric value | Clinically relevant change |
| **TIR^c^ , >70 (%)** |  |  |
|  | 0 | ±10 |
|  | 70 | ±5 |
|  | 100 | ±10 |
| **TBR^d^, <4 (%)** |  |  |
|  | 0 | ±1 |
|  | 4 | ±1 |
|  | 100 | ±10 |
|  |  |  |
| **TBR2^e^, <1 (%)** |  |  |
|  | 0 | ±1 |
|  | 5 | ±1 |
|  | 100 | ±5 |
| **TAR^f^, <25 (%)** |  |  |
|  | 0 | ±5 |
|  | 100 | ±10 |
| **TAR^g^, <5 (%)** |  |  |
|  | 0 | ±5 |
|  | 100 | ±10 |
| **CV^h^, <36 (%)** |  |  |
|  | 0 | ±3 |
|  | 20 | ±3 |
|  | 40 | ±5 |
|  | 100 | ±5 |
| **GMI^i^, <53 (mmol/mol)** |  |  |
|  | 0 | ±2 |
|  | 40 | ±2 |
|  | 70 | ±10 |
|  | 100 | ±10 |

^a^CGM: continuous glucose monitoring.

^b^The commonly used clinical targets of the CGM metrics were used as cutoff values to determine the clinical target errors (εCT). The expert panel boundaries were determined based on expert interviews resulting in clinically relevant changes for different initial values of the CGM metrics. This resulted in the expert panel boundary errors (εEPB).

^c^TIR: time in range.

^d^TBR: time below range.

^e^TBR2: time below range level 2.

^f^TAR: time above range.

^g^TAR2: time above range level 2.

^h^CV: coefficient of variation.

^i^GMI: glucose management indicator.
