# Supplementary material for: Consequences of Data Loss on Clinical Decision-Making in Continuous Glucose Monitoring: Retrospective Cohort Study
Source: Interact J Med Res. 2024 Jul 31;13:e50849. doi: 10.2196/50849 (PMC11325125; doi:10.2196/50849)
Supplement: Multimedia Appendix 3 [file ijmr_v13i1e50849_app3.docx]

The gap probability distribution of the preprocessed CGM data was constructed. If this gap probability distribution follows a fitted exponential probability density function it is missing completely at random (MCAR). The gap probability distribution was cut-off at gaps smaller than 1 day, which is equal to 96 samples. The gap probability distribution that followed was fitted with the exponential probability density function and other common probability density functions with a maximum likelihood estimate (Table 1). Hereafter, the fitted probability density functions were evaluated on goodness of fit with a two-sided Kolmogorov-Smirnov test and the sum of the squared error. The gap probability distribution differed significantly from the exponential probability mass function (*p* < 0.01, Figure 1), but best followed the logarithmic probability mass function (p=0.80 and error of 3.9 × 10^-2^, Table 1). From these results follows that missing data in CGM recordings are not MCAR. Also, most gaps have the length of two samples with a probability of 10.6%.

**Table S1.** The KS-test p-value and sum of the squared error of the fitted exponential and other common probability density functions to the gap probability distribution of all available preprocessed CGM data.

| Name probability density function | p-value from KS-test | Sum of the squared error |
| --- | --- | --- |
| Exponential | <0.01 | 2.9 × 10^-3^ |
| Beta-binomial | 0.01 | 3.7 × 10^-3^ |
| Laplace | <0.01 | 1.1 × 10^-2^ |
| Geometric | <0.01 | 2.9 × 10^-3^ |
| Poisson | <0.01 | 7.3 × 10^-2^ |
| Logarithmic | 0.80 | 3.9 × 10^-2^ |
| Yule-Simon | <0.01 | 7.0 × 10^-2^ |
| Zipfian | <0.01 | 2.0 × 10^-2^ |
|  |  |  |


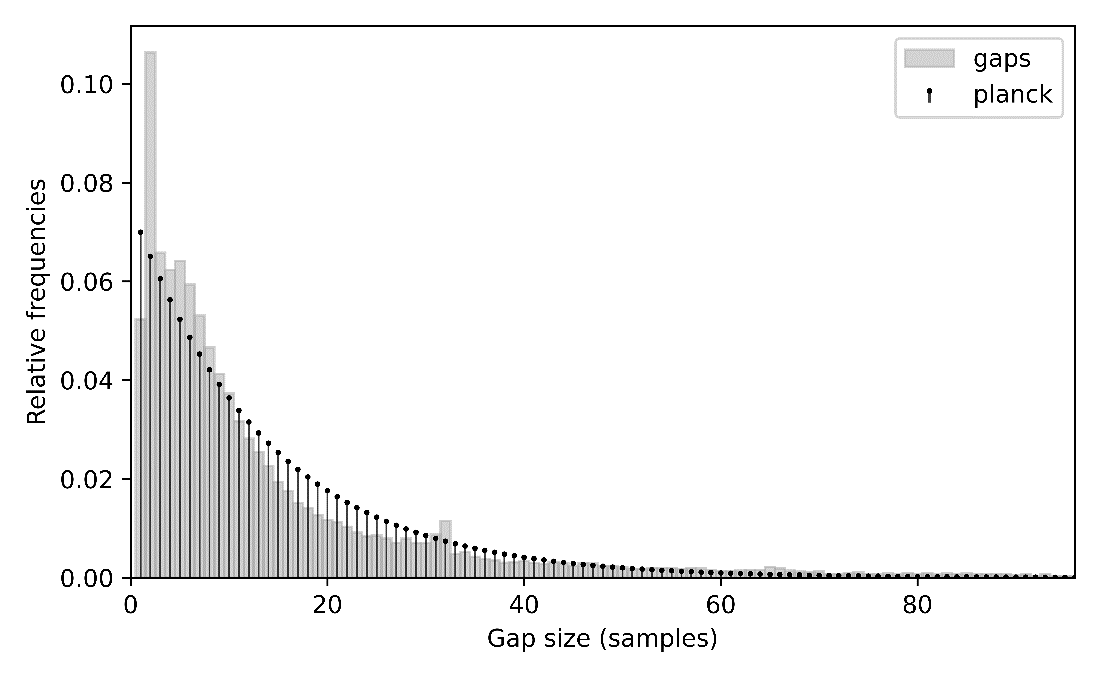


**Figure S1.** The histogram of the gap probability distribution of all available preprocessed CGM data with a fitted exponential probability mass function. Most gaps have the length of two samples (with a probability of 10.6%).
