# Supplementary material for: Consequences of Data Loss on Clinical Decision-Making in Continuous Glucose Monitoring: Retrospective Cohort Study
Source: Interact J Med Res. 2024 Jul 31;13:e50849. doi: 10.2196/50849 (PMC11325125; doi:10.2196/50849)
Supplement: Multimedia Appendix 4 [file ijmr_v13i1e50849_app4.docx]

*Density scatterplots for TIR and CV (Figure S1) and the error plots for glucose metrics TIR, CV, GMI, TAR, TAR2, SD, LBGI, HBGI and RI (Figures S2, S3 and S4).*

*
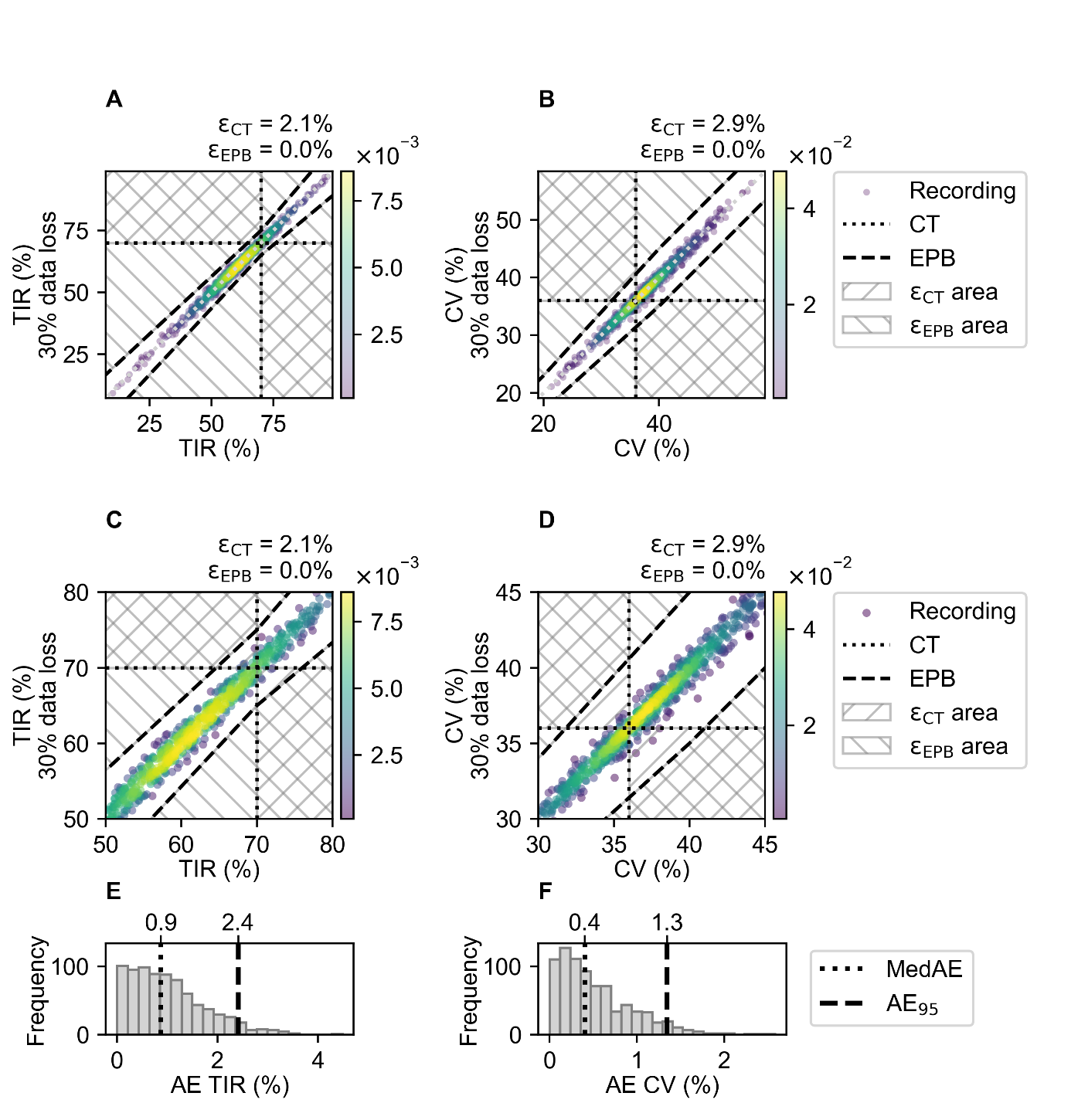
*

*Figure S1–The scatterplots of the time in range (TIR, A), and coefficient of variation (CV, B) indicate the relation between the 14 day long original data without missing data (horizontal axis) and the data with 30% data loss (vertical axis). The color bar indicates the Gaussian kernel-density estimate of the recordings. The black dashed lines represent the expert panel boundaries (EPB), the horizontal and vertical black dotted lines represent the clinical targets (CT). Values falling outside CT and EPB, in the hatched areas, are labeled as errors (ε_CT_ and ε_EPB_). A zoomed in plot is shown in panel C for TIR and CV in panel D. The histograms show the absolute errors (AE, %) with the median AE (MAE) and 95^th^ percentile of AE (AE_95_) as a result of 30% data loss of the time in range (TIR, E), and coefficient of variation (CV, F).*

*
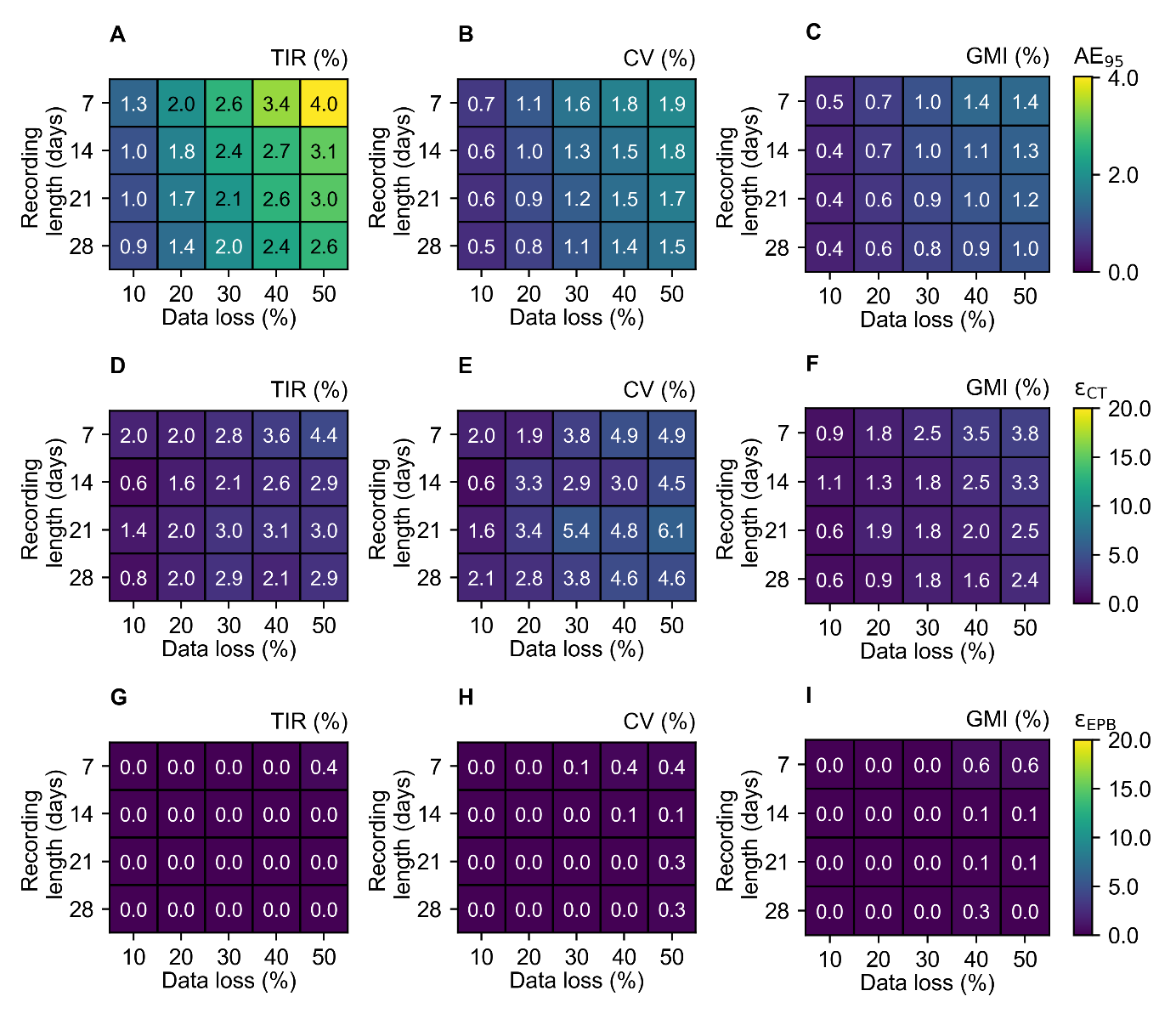
*

*Figure S2–In A–C the 95th percentile of the absolute errors (AE_95_) are shown, in D–F the clinical target errors (ε_CT_), and in G–I the ε_EPB_ of time in range (TIR), coefficient of variation (CV), and glucose management indicator (GMI) respectively of 10, 20, 30, 40 and 50% data loss in recordings of 7, 14, 21 and 28 days. The color bar indicates the errors in percentages.*

*
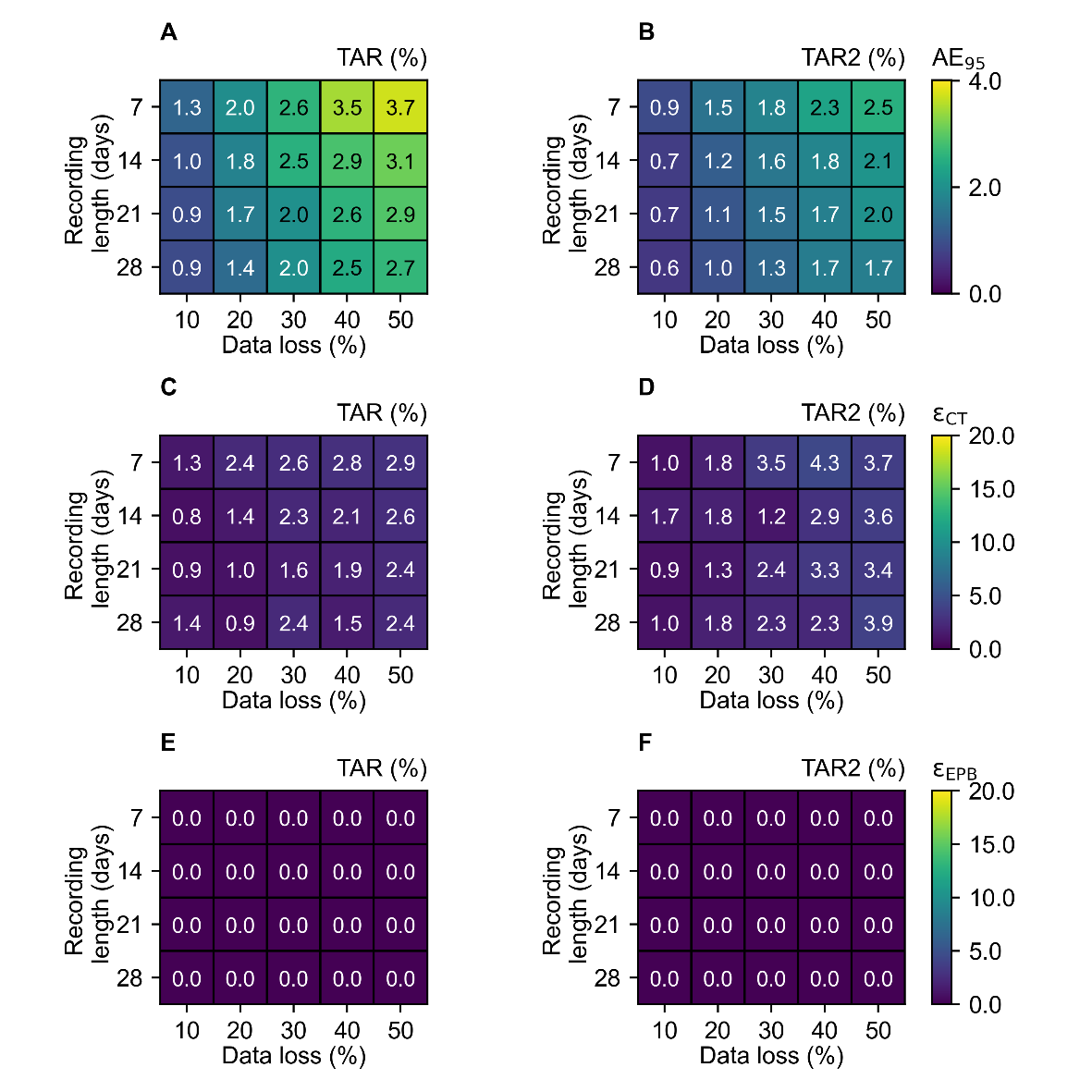
*

*Figure S3–The errors of time above range (TAR), and time above range level 2 (TAR2) as a consequence of 10–50% data loss in recordings of 7–28 days. A and B show the 95th percentile of the absolute errors (AE_95_), C and D the clinical target errors (ε_CT_), and E and F the expert panel boundaries errors (ε_EPB_). The color bar indicates the errors in percentages.*

*
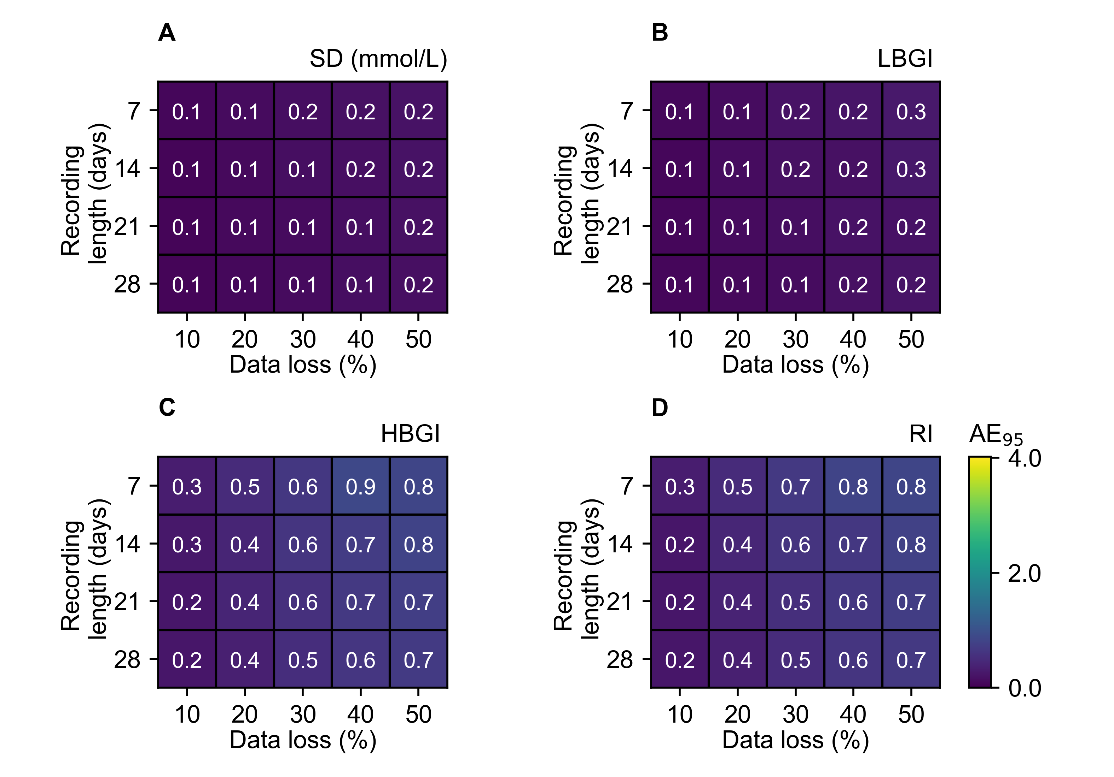
*

*Figure S4–The 95th percentile of the absolute errors (AE_95_) of the standard deviation (SD, A), low blood glucose index (LBGI, B), high blood glucose index (HBGI, C), and risk index (RI, D) as a consequence of 10–50% data loss in recordings of 7–28 days. The color bar indicates the errors in percentages.*
